# Targeted drug delivery with modified gamma-Cyclodextrin nanocarriers and MR-guided focused ultrasound triggering

**DOI:** 10.1186/2050-5736-3-S1-P79

**Published:** 2015-06-30

**Authors:** Doudou Xu, Lijun Wang, Sandy Cochran, Andreas Melzer

**Affiliations:** 1 University of Dundee, Dundee, United Kingdom; 2 Institute for Medical Science and Technology, Dundee, United Kingdom

## Background/introduction

NANOPORATION project sets out to explore specific solutions to overcome the current challenges of targeted drug delivery (TDD) to tumours using magnetic resonance imaging guided focused ultrasound (MRgFUS) to cavitate microbubbles (MBs) for increasing cell permeability and to open ‘drug nano-capsules’ for releasing proven active anticancer drugs directly to the tumour site with reduction of drug dosage needed for the desired therapeutic effect.

## Methods

A novel gamma-Cyclodextrin (gamma-CD) based carrier for encapsulation of doxorubicin (DOX) was synthesized and fully characterized. The encapsulation efficiency was assessed by chemical analysis, *in vitro* and *in vivo*. A high-throughput *in vitro* micro-scale FUS device (sonicator) was designed and applied to cells exposure to carrier-DOX inclusion, in combination with SonoVue^®^ MBs to investigate TDD in monolayer cellular level. *Ex vivo* and *in vivo* trials were carried out by clinical ExAblate MRgFUS system (InSightec, Israel) to establish a safe and efficient clinical TDD protocol on small rodents.

## Results and conclusions

The desired gamma-CD based carrier greatly reduced DOX’s toxicity *in vitro*: up to 95% toxicity reduction in KB human enpidermal carcinoma; up to 92% toxicity reduction in HCT116 colorectal carcinomar. Cellular DOX uptake was reduced 73% in muscle, 69% in kidney, 66% in liver, 65% in heart, 62% in brain, 53% in lungs as 25% in plasma *in vivo*. The carrier-DOX inclusion is highly stable under physiological temperature and pH as well as under a wide range of acidic conditions (pH 1.0~7.0); the encapsulated DOX is slowly released under hyperthermic conditions (up to 50°C). In the presence of MBs (0.1%, 1%, 2.5% and 5%) application of FUS with low mechanical indexes (0.24, 0.31 and 0.53), under which no thermal effect was observed, enhanced up to 3.89-fold of cellular drug uptake for encapsulated DOX *in vitro*. Optimal setup of MR parameters: TR/TE = 3180/96.3msec; bandwidth: 10.4 kHz; Field of View = 20×20cm; matrix: 384×384, NEX: 2; slice thickness: 2.0mm/1.0sp; number of slices: 8; frequency direction: SI and the spatial resolution: 0.52mm; FUS parameters: 4W, 10sec continued sonication and 45sec pulsed sonication with 2.5sec OFF and 0.5sec ON; temperature increase of 7-10 °C; as well as treatment time frame of 35min were identified *ex vivo* and *in vivo*, which allowed application of MRgFUS treatments to live mice bearing tumors under anesthesia with full recovery.

Unfortunately, the lack of detectable DOX signal was obtained from the very first pre-clinical trial. However, the study demonstrated the possibility of translation of the constructed gamma-CD derivative to potential clinical use as a delivery vehicle of DOX for combined thermal and mechanical mechanism by clinically applicable MRgFUS, -triggered TDD for cancer therapy.

**Figure 1:**
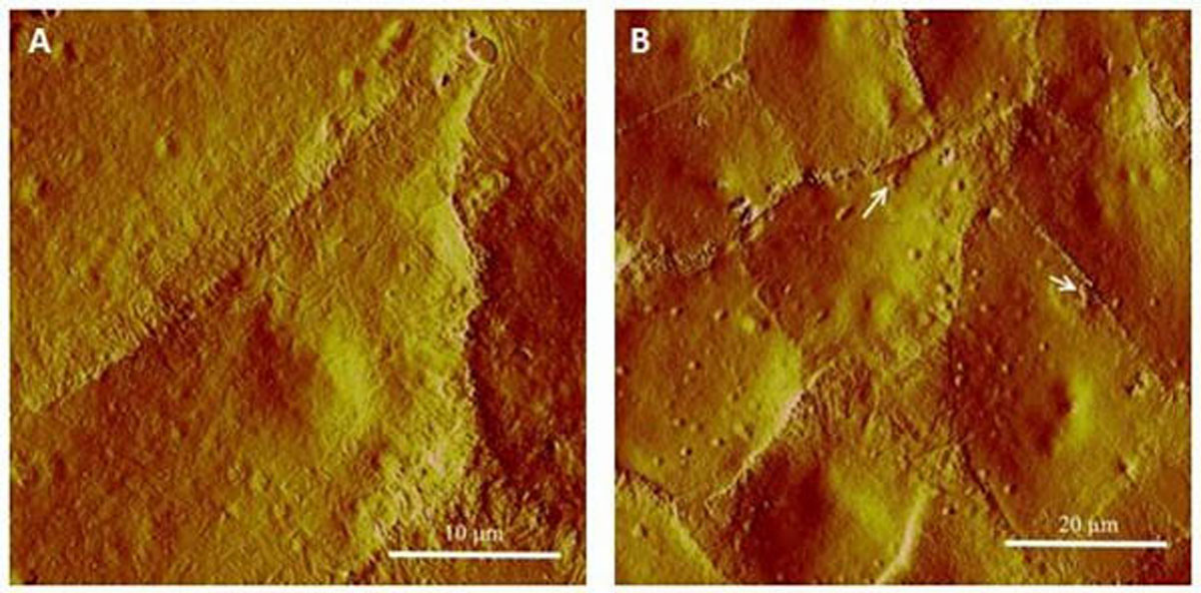
AFM cell surface morphology comparison of KB cells before (A) and after (B) f=0.4868MHz sonication with 2.5% MBs

**Figure 2:**
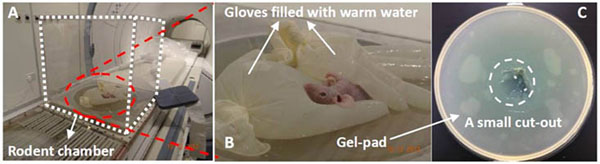
Animal positioning on ExAblate 2000 table inside rodent chamber (A); mouse was protected by gloves filled with water (B); on top of coupling gel-pad with a small cut-out (C)

**Figure 3:**
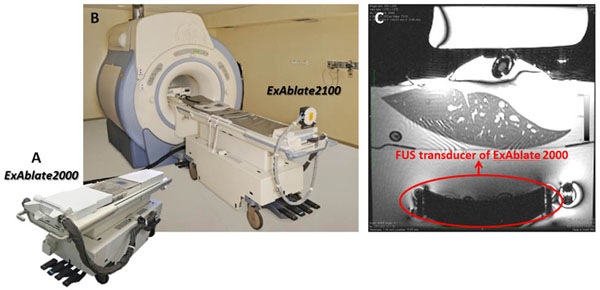
MRgFUS system: ExAblate 2000 (A); ExAblate 2100 with MR (B); a MR image of a sheep liver on top of ExAblate 2000 FUS transducer (C)

**Figure 4:**
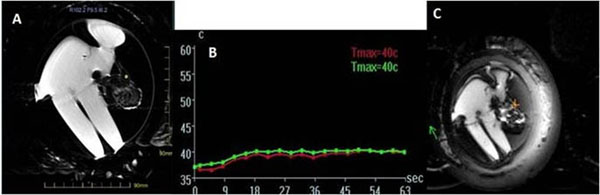
Example of Thermal Monitoring during sonication: Coronal MR image showing planned sonication location (A); temperature graph during sonication (B); the anatomical image acquired during sonication (C)

